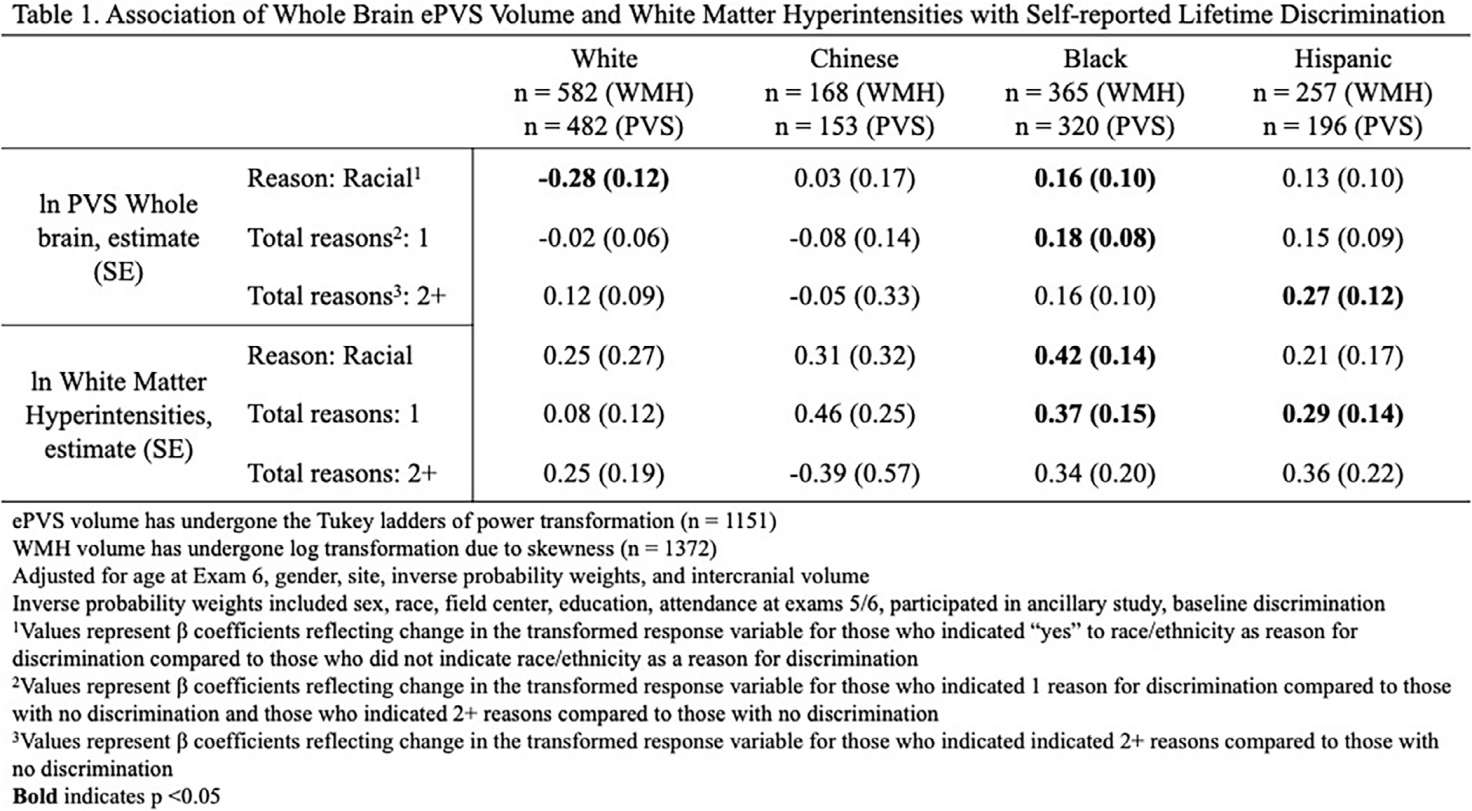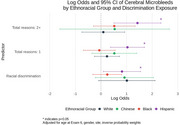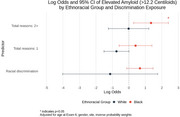# Association of Lifetime Discrimination with Cerebral Small Vessel Disease and Amyloid PET: the Multi‐Ethnic Study of Atherosclerosis (MESA)

**DOI:** 10.1002/alz70862_109860

**Published:** 2025-12-23

**Authors:** Sarah N Forrester, Jordan E. Tanley, Michael P. Bancks, Chinedu T Udeh‐Momoh, Susan R. Heckbert, Mohamad Habes, Stephen R. Rapp, Bonnie C. Sachs, Ilya M. Nasrallah, R. Nick Bryan, Katya Rascovsky, Clara Li, Kathleen M. Hayden, José A Luchsinger, Marcia Pescador Jimenez, Lilah M Besser, Jana A. Hirsch, Wendy Post, Sudarshan Krishnamurthy, Timothy M. Hughes

**Affiliations:** ^1^ University of Massachusetts Chan School of Medicine, Worcester, MA USA; ^2^ Wake Forest University School of Medicine, Winston‐Salem, NC USA; ^3^ Division of Public Health Sciences, Wake Forest University, School of Medicine, Winston‐Salem, NC USA; ^4^ University of Washington, Seattle, WA USA; ^5^ University of Texas Health San Antonio, San Antonio, TX USA; ^6^ Wake Forest School of Medicine, Winston‐Salem, NC USA; ^7^ University of Pennsylvania, Philadelphia, PA USA; ^8^ Icahn School of Medicine at Mount Sinai, New York, NY USA; ^9^ Wake Forest University School of Medicine, Winston Salem, NC USA; ^10^ Columbia University Irving Medical Center, New York, NY USA; ^11^ Boston University School of Public Health, Boston, MA USA; ^12^ University of Miami Miller School of Medicine, Boca Raton, FL USA; ^13^ Drexel University, Philadelphia, PA USA; ^14^ Johns Hopkins Medicine, Baltimore, MD USA

## Abstract

**Background:**

Discrimination impacts various health‐related outcomes and may explain the excess risk for Alzheimer’s disease (AD) and related dementias in minoritized groups through associations with vascular and AD biomarkers related to cognitive function. We hypothesize relationships between discrimation and imaging biomarkers will be observed in minoritized groups.

**Method:**

We used data from MESA, a prospective cohort study that enrolled 6,814 participants at baseline (2000‐02). Exposures were experience of racial discrimination, and number of reasons for discrimination (1 or 2+ reasons) at baseline. During exam periods in 2016‐18 and 2019‐21, brain imaging with 3‐Tesla MRI (*n* = 1372) and amyloid PET (*n* = 422) were completed. The primary outcomes were (1) measures of cerebral small vessel disease including white matter hyperintensities (log WMH, continous), cerebral microbleeds (CMB, present / absent) and enlarged perivascular spaces in the whole brain (log ePVS, continuous); and (2) amyloid PET positivity (Centiloids ≥ 12.2). Multivariable linear (WMH and ePVS) and logistic (CMB and amyloid PET positivity) regression models were adjusted for covariates including age, sex, site, education, and inverse probability weight. WMH and ePVS models were also adjusted for intracranial volume. All models were stratified by ethnoracial group.

**Result:**

Participants were 72.3 (7.8) years of age on average at MRI and were 42% White, 11% Chinese, 26% Black, and 21% Hispanic. Black and Hispanic participants were more likely than other groups to report experiencing racial discrimination and a higher burden (2+ reasons) of discrimination. Racial discrimination was significantly associated with smaller ePVS volumes in White participants, and larger ePVS volumes in Black participants, endorsing 2+ reasons for discrimination was associated with larger ePVS volumes in Hispanic participants. Reporting one reason for discrimination was associated with higher volume of WMH in Black and Hispanic particpants (Table 1). Lifetime discrimination was associated with higher odds of CMB in Hispanic participants (Figure 1). Endorsing 2+ reasons was significantly associated with higher burden of cerebral amyloid among Black participants (Figure 2).

**Conclusion:**

Experiences of lifetime racial discrimination and burden of various forms of lifetime discrimination are associated with imaging biomarkers implicated in cognitive function. Associations are more pronounced within different ethnoracial groups.